# Pharmacokinetic/Pharmacodynamic Target Attainment of Ceftazidime in Adult Patients on General Wards with Different Degrees of Renal Function: A Prospective Observational Bicenter Cohort Study

**DOI:** 10.3390/antibiotics12030469

**Published:** 2023-02-25

**Authors:** Saskia E. Zieck, Suzanne L. de Vroom, Frouke Ph. Mulder, Gitte van Twillert, Ron A. A. Mathôt, Suzanne E. Geerlings, Reinier M. van Hest

**Affiliations:** 1 Department of Pharmacy and Clinical Pharmacology, Amsterdam UMC, University of Amsterdam, Meibergdreef 9, 1105 AZ Amsterdam, The Netherlands; 2 Department of Hospital Pharmacy, Division of Clinical Pharmacology, Noordwest Ziekenhuisgroep, Location Alkmaar, Wilhelminalaan 12, 1815 JF Alkmaar, The Netherlands; 3 Department of Internal Medicine, Division of Infectious Diseases, Amsterdam UMC, University of Amsterdam, Meibergdreef 9, 1105 AZ Amsterdam, The Netherlands; 4 Department of Internal Medicine, Division of Infectious Diseases, Noordwest Ziekenhuisgroep, Location Alkmaar, Wilhelminalaan 12, 1815 JF Alkmaar, The Netherlands

**Keywords:** ceftazidime, pharmacokinetics, renal impairment, target-attainment, general wards

## Abstract

No prospective evidence exists on the pharmacokinetic/pharmacodynamic (PK/PD) target attainment of ceftazidime in adult patients on general wards. We aimed to investigate whether the PK/PD target of ceftazidime (50% T > MIC) is attained in adult patients on general wards with adequate and impaired renal function receiving regular and guideline-recommended reduced doses of ceftazidime. In this observational, prospective, bicenter cohort study, adult patients admitted to a general ward receiving ceftazidime as part of standard care were included. Three blood samples per patient within 72 h after start of treatment were collected. Data were analyzed with nonlinear mixed effects modeling. The primary endpoint was target attainment of 50% T > MIC during the first 24 h of treatment (50% T_0–24_ > MIC). Forty patients were included from whom 121 blood samples were obtained. All 25/25 patients with adequate renal function, 9/10 patients with moderately impaired renal function (eGFR 30–50 mL/min/1.73 m^2^) and 5/5 patients with severe impaired renal function (eGFR < 30 mL/min/1.73 m^2^) attained 50% T_0–24_ > MIC when applying the clinical breakpoint MIC for *Pseudomonas aeruginosa* of 8 mg/L. The one patient not attaining the PK/PD target did not differ in any of the collected patients’ characteristics, except that this patient was the oldest in the study population. However, age was not statistically significantly associated with clearance or volume of distribution in the population pharmacokinetic model and, therefore, not likely the cause for this patient not attaining the PK/PD target. Our results suggest ≥90% probability of the PK/PD target attainment of ceftazidime in patients on general wards with adequate and impaired renal function receiving regular and guideline-recommended reduced doses of ceftazidime for treatment of infections with *Pseudomonas aeruginosa* and all bacteria with lower MIC-values.

## 1. Introduction

Appropriate and early antibiotic treatment are primary determinants of mortality in patients with bacterial infections [1,2,3]. Antibiotic treatment is considered to be appropriate when the relevant pharmacokinetic/pharmacodynamic (PK/PD) target is attained. Optimal antibiotic dosing regimens aiming to attain those PK/PD targets are, therefore, of high importance to prevent treatment failure [4,5].

The third-generation cephalosporin antibiotic ceftazidime is frequently administered to hospitalized patients with various infections caused by Gram-negative bacteria, particularly *Pseudomonas aeruginosa* (*P. aeruginosa*) [6]. Ceftazidime exhibits, like other beta-lactams, time-dependent killing [7,8,9,10]. Therefore, successful treatment outcomes in terms of bacterial eradication and clinical cure is associated with the percentage of time of the dosing interval that the serum concentration remains above the minimum inhibitory concentration (T > MIC) [7,8,9,10]. The MIC is defined as the lowest concentration of an antibiotic that prevents visible growth of bacteria in vitro [11]. For ceftazidime, the T > MIC value needed for bactericidal activity is reported to be between 30% and 100%. A target of 50% T > MIC is the most commonly used target and best associated with clinical efficacy in patients admitted to general wards [7,8,9,10].

Ceftazidime shows low protein binding of 10% and is almost exclusively eliminated through the kidneys [12,13]. Consequently, a dose reduction is recommended for patients with impaired renal function [12,13,14]. However, physicians do not apply this dose reduction in half of all patients with impaired renal function, although a dose reduction is recommended by the applicable guideline [15]. A cause of this might be that currently advised dose reductions are merely based on extrapolations of small studies investigating a full, unadjusted, dose of ceftazidime [16,17,18,19,20]. Only one study investigated steady-state pharmacokinetics in patients with renal impairment receiving a reduced dose; however, only critically ill patients were included with concomitant use of furosemide [20].

Although a variety of studies have been conducted to assess the PK/PD target attainment of ceftazidime in critically ill patients, we have not identified such studies in adult patients on general wards as these patients are likely to exhibit other pharmacokinetics compared to critically ill patients [16,17,18,19,20,21]. As such, no prospective evidence exists that currently guideline-recommended ceftazidime dosing regimens result in at least 50% T > MIC in adult patients on general wards, especially not in patients with renal impairment receiving a reduced dose of ceftazidime. Therefore, the aim of this study is to determine the probability of attaining the PK/PD target (PTA) of ceftazidime (50% T > MIC) in the first 24 h of treatment in adult patients on general wards with adequate and impaired renal function receiving regular and guideline-recommended reduced doses of ceftazidime.

## 2. Results

### 2.1. Patients and Ceftazidime Concentrations

Forty patients were included of which there were twenty-five patients with adequate renal function (eGFR ≥ 50 mL/min/1.73 m^2^), ten patients with moderate renal impairment (eGFR 30–49 mL/min/1.73 m^2^) and five patients with severe renal impairment (eGFR 10–29 mL/min/1.73 m^2^). All patients were treated with the guideline-recommended dose of ceftazidime based on their renal function (Table 1), except for one patient with severe renal impairment that was treated with 2000 mg q24h instead of the recommended 1000 mg q24h. We decided to keep this patient in the dataset for analysis of the primary outcome as this patients’ ceftazidime level at 12 h after start of therapy was well above the worst-case MIC of 8 mg/L (namely 24.3 mg/L), making it highly likely that 50% T > MIC within the first 24 h of treatment would have been attained if 1000 mg would have been administered. Age, serum creatinine, eGFR and the department of admission differed significantly between the three renal function groups (Table 2).

A total of 121 samples were collected of which 52 samples (43%) were obtained within the first 24 h of treatment. Two samples were excluded. The first excluded sample was collected at the same time point in the same patient as another sample and, therefore, did not add additional information for population PK analysis. The second excluded sample was a sample with a ceftazidime concentration < lower limit of quantification (LLOQ) that followed a sample from the same patient that also already was <LLOQ. This left a total of 119 samples of which 1 was <LLOQ and none > upper limit of quantification (ULOQ) for nonlinear mixed effects modeling (NONMEM). Five of these samples were obtained from waste material. From five patients (12.5%) only two samples per patient could be drawn.

### 2.2. Population Pharmacokinetic Analysis

A one-compartment model with first-order elimination best described the pharmacokinetics of ceftazidime (Table 3). The interpatient variability (IIV) of ceftazidime could be estimated for clearance (CL) and volume of distribution (V). During the multivariate analysis, a statistically significant association (*p* < 0.01) was found between eGFR (CKD-EPI) and CL, which explained a large part of the IIV in CL: IIV CL drop from 78.9% to 37.6% upon inclusion of this association. Furthermore, an association was found between the concomitant use of antibiotics and CL. This association also explained some IIV in CL as IIV CL decreased from 37.6% to 31.3% upon inclusion of this association (Table 3). There was no missing covariate data in the dataset.

**Figure 1:**
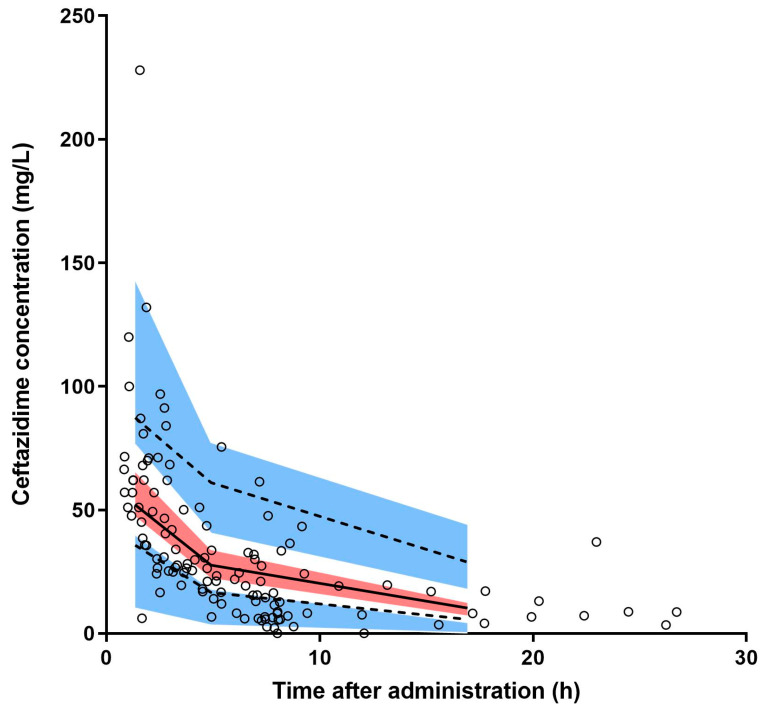
Prediction corrected visual predictive check of the final model. The dots represent the prediction corrected observed ceftazidime concentrations. The solid black line represents the observed median and the dashed black lines represent the 5th and 95th percentiles of the observed prediction-corrected data. The blue areas represent the 95% confidence interval of the model-predicted 5th and 95th percentiles. The red area represents the 95% confidence interval of the model-predicted median. The solid and upper dashed black lines run within their respective shaded areas, thus showing that the model adequately predicts the observed data. The lower dashed black line rises slightly above the blue shaded area at the end of the dosing interval, indicating a slight underestimation of the observed 5th percentile. Overall, this VPC demonstrates a sufficient fit of the final model.

The goodness of fit plots (GOF) (Supplementary Material Figure S1) and the prediction corrected visual predictive check (VPC) (Figure 1) show that the final model is able to adequately describe the observed ceftazidime concentrations and was, therefore, valid to be used to calculate individual T > MIC and AUC values. The NONMEM control stream of the final model can be found in Supplementary File S1.

### 2.3. Pharmacokinetic/Pharmacodynamic Target Attainment

For an MIC of 8 mg/L, which is the clinical breakpoint of *P. aeruginosa*, the probability of PK/PD target attainment of 50% T > MIC within the first 24 h of treatment (the primary outcome) was 100% (25/25) in patients with adequate renal function receiving the regular dose, 90% (9/10) in patients with moderate renal impairment receiving a reduced dose and 100% (5/5) in patients with severe renal impairment receiving a reduced dose (Table 4). For the secondary outcomes, the patient with severe renal impairment due to receiving a significantly different dose (2000 mg q24h instead of 1000 mg q24h) was excluded. For an MIC of 8 mg/L PTA of 100% T_0–24_ > MIC was 24% (6/25) in patients with adequate renal function receiving the regular dose, 50% (5/10) in patients with moderate renal impairment receiving a reduced dose and 75% (3/4) in patients with severe renal impairment receiving a reduced dose (Table 5). For the secondary endpoint, PTA of 50% T_24–48_ > MIC, a second patient was excluded, namely one patient with adequate renal function who received ceftazidime therapy during only the first 24 h of treatment. The PTA of 50% T_24–48_ > MIC for an MIC of 8 mg/L was 100% (24/24) in patients with adequate renal function, 90% (9/10) in patients with moderate renal impairment and 100% (4/4) in patients with severe renal impairment (Table 6). 

### 2.4. Monte Carlo Dosing Simulations

**Figure 2:**
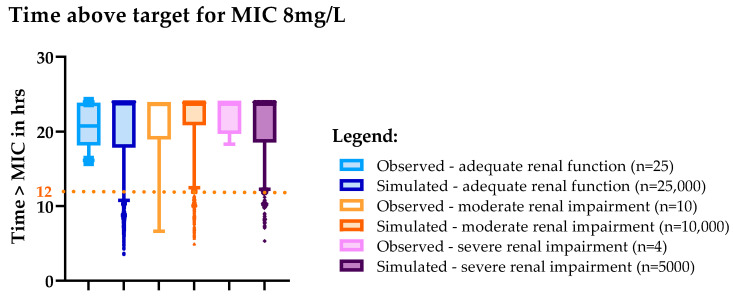
Boxplots of observed and simulated time above target for MIC 8 mg/L for patients with adequate, moderately impaired and severely impaired renal function within the first 24 h of treatment. Presented are the median (horizontal line within the box), the interquartile range (box) and the 5th and 95th percentile (whiskers). The target for 50% T > MIC_0–24_ is presented as the orange dotted line at 12 h.

The majority of the study population (n = 22, 55%) received more ceftazidime administrations during the first 24 h of treatment than prescribed due to the fact that follow-up administrations were planned during the routine time windows of nurses’ administration rounds. This often resulted in drug administration early in the morning following the day of ceftazidime initiation. From that moment on, the dosing interval as prescribed was more accurately adhered to. As a consequence, the time above MIC in the first 24 h for these individuals is higher than when perfect dosing intervals of 8, 12 or 24 h (depending on renal function) would have been applied after the first dose. To examine the influence of this phenomenon on the PTA, the original dataset, but then with exact dosing intervals of q8, q12 or q24 depending on the renal function, was simulated 1000 times by means of a Monte Carlo simulation with the final model. The PTA of 50% T_0–24_ > MIC remained high: 93%, 97% and 97% for patients with adequate, moderately impaired and severely impaired renal function, respectively (Figure 2). A similar PTA was found in the simulated patients when compared to the PTA as observed in the included patients indicating minimal bias in PTA of 50% T_0–24_ > MIC due to the dose shift in our study population. 

### 2.5. Drug Exposure

**Figure 3:**
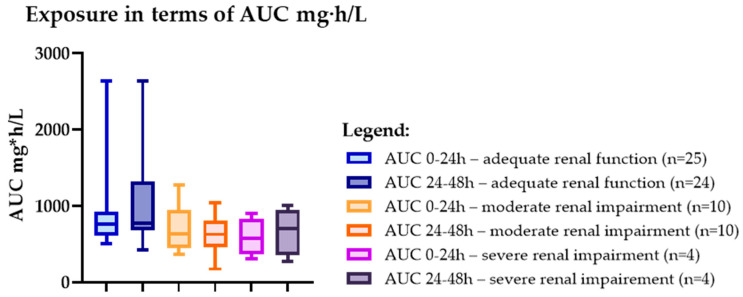
Exposure to ceftazidime in terms of AUC (mg∙h/L) for the first 24 h of treatment (AUC_0–24_) and the second 24 h of treatment (AUC_24–48_) for different renal function groups. AUCs >1500 mg∙h/L in the adequate renal function group were patients with eGFRs ranging between 51 and 77 mL/min/1.73 m^2^.

No differences in median drug exposure in the first 24 h of treatment (AUC_0–24_) and 24–48 h after start of treatment (AUC_24–48_) were observed between patients with adequate renal function receiving regular doses and patients with moderately impaired and severely impaired renal function receiving the guideline-recommended reduced doses (*p* = 0.159 and *p* = 0.125) (Figure 3).

### 2.6. Clinical Outcome Measure

Since only one out of 40 patients (2.5%) did not attain the primary outcome of 50% T_0–24_ > MIC for MIC values up to 8 mg/L, we did not explore clinical outcome in this study since a minimum of 25%, or 10 patients, not attaining the primary outcome was a prerequisite for exploring clinical outcome.

## 3. Discussion

This study shows that with the current dosing regimen of ceftazidime, the PTA of 50% T_0–24_ > MIC is attained in ≥90% of adult patients on general wards treated for clinically relevant bacteria with MICs ≤8 mg/L. Therefore, the current dosing regimen proves adequate treatment of infections caused by *P. aeruginosa*, which have a clinical breakpoint of 8 mg/L as defined by the EUCAST [22].

No differences in PTA or drug exposure were observed between patients with adequate renal function receiving regular doses versus patients with moderately impaired and severely impaired renal function receiving the guideline-recommended reduced doses. This is in contradiction with the results from a comparable study with ciprofloxacin, which did show differences in drug exposure and PTA between patients with adequate renal function receiving regular doses and patients with impaired renal function receiving a 50% dose reduction [23]. This phenomenon may be explained by the fact that ceftazidime is eliminated exclusively through the kidneys, so no compensating pathways for excretion through the hepatic system exist as is the case for ciprofloxacin. Therefore, a gross dose reduction in case of renal impairment, as currently recommended and investigated in this study, seems appropriate. 

One patient with moderate renal impairment showed a serum concentration of ceftazidime below the LLOQ at 8.02 h after ceftazidime administration. This was the only patient not attaining the PK/PD target. All collected characteristics of this patient were compared with the characteristics of the included population. No ‘end of the spectrum’ patients’ characteristics were observed in this patient, except for age. This patient was 86 years old, which was the oldest age observed in our population. However, we have tested the covariate age in our population pharmacokinetic model and age was not statistically significant associated with CL or V. Therefore, oldest age is not likely the cause for this patient not attaining the PK/PD target and the cause of this low exposure remains unknown. However, the PTA remained ≥90% within the moderate renal function group, which was defined as the minimum acceptable PTA. 

In the present study, a one-compartment population PK model of ceftazidime was developed with associations between eGFR (CKD-EPI) and CL and between concomitant use of antibiotics and CL. This model could predict our observed data sufficiently well as seen in the visual predictive check (Figure 1). The association between the eGFR (CKD-EPI) and CL was as expected [24,25]. The statistically significant association between CL and the concomitant use of other antibiotics was unexpected. Although we could not find a physiological explanation for this association, we decided to retain this association within the final model as it gave a statistically significant drop of the objective function during the multivariate analysis (*p* < 0.01), the corresponding parameter quantifying the effect of concomitant use of other antibiotics on CL was precisely estimated (Table 3) and it explained 16.8% IIV in CL. Nevertheless, it cannot be ruled out that the identification of this association is based on coincidence. 

This is, to the best of our knowledge, the first prospective study measuring PK/PD target attainment of ceftazidime in real-life clinical practice in patients on general wards. The developed population PK model that was used for the calculation of the outcome parameters T > MIC and AUC values showed low residual variability, indicating careful collection of study data. Additionally, this study was conducted in an academic medical center and a peripheral teaching hospital and included patients that were admitted to a broad variety of wards (e.g., cardiology, hematology, internal medicine, nephrology, orthopedic surgery and respiratory department), enhancing the representativeness of the included patient population. 

Nevertheless, several limitations of this study should also be considered. First, a shift was observed in timepoint of drug administration in the morning following the day of ceftazidime initiation. As a result, more than half of our included patients (n = 22) received an additional antibiotic administration during the first 24 h of treatment than originally prescribed due to the fact that follow-up administrations are planned during the routine time windows of nurses’ administration round. This is inherent to the observational design of this study and may well lead to a higher PTA than when exact dosing intervals as prescribed would have been applied. However, Monte Carlo simulations were performed using exact dosing intervals. Results showed comparable PTA. Additionally, this dosing shift is representative for real-life clinical practice. 

Second, one patient with severe renal impairment received a dose of 2000 mg q24h, that differed from the regular renal function-based dose adjustment within this group of 1000 mg q24h. Therefore, T > MIC is overestimated in this patient. Yet, the estimated concentration in this patient at t = 12 h after the first ceftazidime administration of 2000 mg was 24,3 mg/L, which makes it reasonable to assume that the concentration would also be >8 mg/L at t = 12 h if 1000 mg would have been administered, assuming that a factor two lower dose will grossly lead to a factor two lower concentration at the same time after the first administration. With this assumption, >50% T_0–24_ > MIC would have been obtained with 1000 mg q24 h. We, therefore, decided not to exclude this patient from our analysis of the primary outcome. We did exclude this patient for all secondary outcomes because these are obviously overestimated with the higher dose and because no simple and reasonable assumption, as for the primary outcome, could be made with regard to AUC values and attainment of 100% T_0–24_ > MIC if 1000 mg would have been administered, since a second dose of 2000 mg was already administered 18 h after the first one with an estimated trough level of 14 mg/L.

Third, the collected number of samples (121 of which 2 were excluded) is quite small, limiting the possibility to identify, e.g., a two-compartmental model or to identify more covariate associations. 

Physicians are hesitant to adjust the dose of antibiotics in cases of renal impairment, probably due to fear of insufficient exposure [26,27,28]. For the antibiotic ciprofloxacin, our group has previously shown that this fear seems justified [16]. This research adds valuable evidence regarding the currently advised dosing regimen of ceftazidime used to treat patients with moderately impaired and severely impaired renal function admitted to general wards as we show that dose adjustment of ceftazidime in renal impairment results in adequate PTA and comparable exposure in comparison with patients with adequate renal function receiving 2000 mg q8h. Any potential fear among prescribers for underdosing thus appears to be unfounded.

## 4. Materials and Methods

### 4.1. Study Design

This prospective, bicentre, observational cohort study was conducted between October 2019 and December 2021 on general wards at the Amsterdam UMC—location AMC (AMC); or Noordwest Ziekenhuisgroep—location Alkmaar (NWZ).

This study was performed according to the principles of the Declaration of Helsinki (October 2013) and in accordance with the Medical Research Involving Human Subjects Act (WMO) [29,30]. The study was approved by the ethics committee of Amsterdam UMC—location AMC. Patients participating in this study all signed written informed consent before inclusion.

### 4.2. In- and Exclusion Criteria

Patients were eligible when meeting the following inclusion criteria: (1) adult patients (age ≥ 18 years); (2) admitted to a general ward of AMC or NWZ; and (3) receiving therapeutic dosages of ceftazidime as part of standard care prescribed by their attending physician and according to the current local guideline, which is adapted from the national antimicrobial guide [12,13,14]. Patients were excluded if: (1) written informed consent was not obtained; (2) a patient was mentally incapacitated; or (3) patients with known altered pharmacokinetics compared to patients on general wards, i.e., patients in the ICU, patients undergoing renal replacement therapy, patients with cystic fibrosis and patients with severe burns [31,32,33].

### 4.3. Sample Size Calculation

Since no data were available in the literature on the percentage of patients with impaired renal function attaining 50% T_0–24_ > MIC to base the sample size calculation on, we based our sample size calculation on the second-best available data, namely detecting an association between renal function and clearance of ceftazidime. Detection of such an association is a prerequisite for analysing differences in target attainment of ceftazidime between populations with adequate and impaired renal function. A Stochastic Simulation and Estimation (SSE) procedure as implemented in the Pearl Speaks NONMEM software (version 3.5.3, Uppsala, Sweden) was applied for this purpose. In this Monte Carlo simulation procedure, the two-compartment population pharmacokinetic model as described by Delattre et al. was used [34]. A blood sample collection scheme of 3 samples per patient (1 trough and 2 random samples) within a total sample size of 40 patients was shown to have a power of >95% with an alpha level of 0.05 to detect an association between renal function and ceftazidime clearance. A total of 15 of the 40 patients needed to be included with an estimated glomerular filtration rate (eGFR) ≤ 50 mL/min/1.73 m^2^, of whom at least 5 had an eGFR < 30 mL/min/1.73 m^2^.

### 4.4. Study Procedure

Dose and duration of ceftazidime treatment were determined by the discretion of the attending physician. The recommended dosing regimen for patients with adequate renal function varies between guidelines, but in general the dose is 500 mg every 12 h (q12h) to 2000 mg every 8 h (q8h) is recommended. In this study a dosing regimen of 2000 mg q8 was investigated in accordance with the local antimicrobial guidelines of the participating hospitals [12,13,14]. Based on (inter)national and local guidelines, the dose of ceftazidime is adjusted when eGFR is 30–50 mL/min/1.73 m^2^ to 1000 mg q12h and when eGFR is below 30 mL/min/1.73 m^2^ to 1000 mg every 24 h (q24h) [12,13,14].

Demographic data, clinical data, laboratory data (e.g., serum creatinine and renal function expressed as eGFR (CKD-EPI)) of included patients, as well as the administration data of ceftazidime were derived from the electronic patient record and were stored anonymized in an online database subsumed into Castor EDC. 

Preferably within 24 h but at least within 72 h after the start of ceftazidime treatment, three blood samples, one trough level and two random samples were prospectively collected in a vacutainer tube without anticoagulant for ceftazidime concentration measurement. Blood samples were immediately centrifuged at 3000× *g* after sample collection and the plasma was stored frozen at −80 °C until analysis. As part of the study protocol, waste material of samples obtained for standard care during ceftazidime treatment were, if available, collected from the Laboratory of Clinical Chemistry of the AMC and NWZ. Determination of total ceftazidime plasma concentration in the obtained blood samples was performed at the laboratory of the Department of Hospital Pharmacy & Clinical Pharmacology of the AMC, using a validated high-performance liquid chromatography tandem mass spectrometry (LC-MS/MS) method (LC30 Shimadzu, Kyoto, Japan; MS QTRAP 5500 system, SCIEX, Framington, Massachusets, United States of America). The method had a lower limit of quantification (LLOQ) of 0.1 mg/L and an upper limit of quantification (ULOQ) of 40 mg/L. In case concentrations above the ULOQ were measured, the sample was diluted and reanalyzed. In case concentrations below the LLOQ were measured, the ceftazidime concentration in the sample was set to a 0.5-fold lower concentration than the LLOQ for data analysis. The accuracy of the method at the LLOQ (0.1 mg/L) and ULOQ (40 mg/L) was 117% and 106%, respectively. The precision of the method at the LLOQ (0.1 mg/L) and ULOQ (40 mg/L) were below 3.86% and 1.62%, respectively.

### 4.5. Study Outcomes

The primary outcome of this study was target attainment defined as a ceftazidime concentration that exceeded the MIC during more than 50% (i.e., >12 h) of the first 24 h of IV treatment (50% T_0–24_ > MIC). This parameter was subsequently used to calculate the probability of target attainment during the first 24 h of treatment, which was defined as the percentage of patients that attained 50% T_0–24_ > MIC. The PTA was calculated for patients with adequate, moderately impaired and severely impaired renal function receiving regular and guideline-recommended reduced doses of ceftazidime. An MIC of 8 mg/L was considered most important, being the clinical breakpoint MIC of *P. aeruginosa* for ceftazidime and, therefore, the highest breakpoint of all ceftazidime-susceptible microorganisms and the microorganism that usually needs to be covered when treating with ceftazidime [6]. A PTA of ≥90% was considered adequate.

Secondary outcomes were target attainment of 50% T > MIC between 24 and 48 h of treatment (50% T_24–48_ > MIC) and target attainment of 100% T > MIC during the first 24 h of treatment (100% T_0–24_ > MIC), both for the calculation of PTA for these targets. In this case, 100% T_0–24_ > MIC was defined as 23.5 h of the first 24 h above the MIC given the infusion time of the first dose was 0.5 h; therefore, the ceftazidime concentration will be below the MIC for at least a part of this infusion time. A further secondary outcome was area under the concentration–time curve (AUC) at 24 h and 24–48 h after start of treatment (AUC_0–24_ and AUC_24–48_) to compare ceftazidime exposure. All primary and secondary outcomes were calculated for the three different renal function groups:

- Group I: adequate renal function; eGFR ≥ 50 mL/min/1.73 m^2^ treated with regular doses of ceftazidime (2000 mg q8).
- Group II: moderately impaired renal function; eGFR 30–49 mL/min/1.73 m^2^ treated with reduced doses of ceftazidime (1000 mg q12).
- Group III: severely impaired renal function; eGFR 10–29 mL/min/1.73 m^2^ treated with reduced doses of ceftazidime (1000 mg q24).

If a large proportion, defined as a percentage of 25% or a minimum of 10 patients, does not attain the primary outcome of 50% T_0–24_ > MIC, we will explore whether or not attaining this target is associated with patients’ clinical outcome.

### 4.6. Statistical Analysis & Pharmacokinetic Model

The data in this study are presented as frequencies (categorical data) and median values (continuous data) with the interquartile range (IQR). Differences between groups were calculated for continuous values using the Kruskal–Wallis test and for categorical data using the Pearson Chi-square test with IBM-SPSS v28 (IBM corporation, Armonk, New York, USA). Differences were considered statistically significant at a *p*-value of <0.05.

A compartmental population pharmacokinetic model for ceftazidime was developed using non-linear mixed effects modelling (NONMEM) (v7.5 Icon Development Solutions, Ellicot City, Maryland, USA) to be able to calculate T > MIC and assess target attainment. The model was parameterized using the primary pharmacokinetic parameters volume of distribution (V) and clearance (CL). First, a structural model was developed by testing one and two compartmental models as well as interpatient variability (IIV) and interoccasion variability (IOV) in the pharmacokinetic parameters. Afterwards, a covariate analysis was performed in which demographic and pathophysiological data of the included patients were tested for their association with CL and V with first a univariate analysis and subsequently a multivariate analysis with all statistically significantly associated covariates from the univariate analysis. This resulted in the final model. The following covariates were tested: serum creatinine, eGFR calculated with CKD-EPI formula, eGFR calculated with the Modification of Diet in Renal Disease (MDRD) formula, eGFR calculated with the Cockroft and Gault formula, BMI, age, ethnicity, admission to the orthopedic ward, admission to the hematology ward, fever yes/no (‘yes’ defined as body temperature >38°) and concomitant use of other antibiotics. The fit of the model was evaluated using goodness-of-fit plots, the objective function and precision of the parameter estimates. The robustness and internal validity of the model was tested with a bootstrap analysis (n = 1000) and a prediction corrected visual predictive check (VPC). The T > MIC for each individual patient was estimated using the empirical Bayesian estimates of the pharmacokinetic parameters from the final and internally validated model with which subsequently target attainment per patient could be assessed. Also, AUC_0–24_ and AUC_24–48_ for each individual patient was estimated using the empirical Bayesian estimates of the pharmacokinetic parameters from the final model.

## 5. Conclusions

In conclusion, the PTA of ceftazidime of 50% T_0–24_ > MIC is ≥90% in adult patients on general wards with adequate and impaired renal function receiving regular and guideline-recommended reduced doses of ceftazidime for the treatment of clinically relevant bacteria with MICs ≤ 8 mg/L. Therefore, the current dosing regimens for both patient categories are adequate for the treatment of infections caused by *P. aeruginosa*, which have a clinical breakpoint of 8 mg/L as defined by the EUCAST [22]. 

## Figures and Tables

**Table 1:** Guideline-recommended dose of ceftazidime.

Renal Function Group	Guideline-Recommended Dose of Ceftazidime
Adequate renal function (eGFR ≥ 50 mL/min/1.73 m^2^, (n = 25))	2000 mg q8h
Moderate renal impairment (eGFR ≥ 30–50 mL/min/1.73 m^2^, (n = 10))	1000 mg q12h
Severe renal impairment (eGFR < 30 mL/min/1.73 m^2^, (n = 5))	1000 mg q24h

**Table 2:** Baseline characteristics (n = 40) of the included patient population. Patients were classified according to their renal function expressed as eGFR (CKD-EPI) on the day of inclusion.

Variable *	Overall	eGFR ^a^	eGFR ^a^	eGFR ^a^
		≥50 mL/min/1.73 m^2^	≥30–50/1.73 m^2^	<30 mL/min/1.73 m^2^
	n = 40	n = 25	n = 10	n = 5
Female, n	17	9	6	2
Age, yrs	62.0	56.0 (40.5–68.5)	72.0	64.0
	(47.0–72.0)		(69.8–86.0)	(41.5–73.0)
Weight, kg	79.6	80.0	78.5	71.7
	(69.7–92.3)	(71.8–89.0)	(67.2–94.3)	(57.1–140.6)
Height, cm	175.5	180.0	171.5	167.0
	(167.0–185.0)	(168.0–185.0)	(163.5–184.3)	(163.0–179.5)
BMI, kg/m^2^	25.0	24.7	26.2	23.4
	(22.0–29.0)	(21.3–27.8)	(23.7–31.4)	(21.3–46.5)
Ethnicity				
*Caucasian*	32	22	8	2
*African American*	4	1	2	1
*Asian*	3	1	0	2
*Hispanic*	1	1	0	0
Serum creatinine	100.0	72.0	135	328.0
	(67.3–135.3)	(59.5–92.5)	(118–162)	(217.5–430.0)
eGFR ^a^, mL/min/1.73 m^2^	73.5	102.8	34.3	18.6
	(34.3–111.4)	(78.1–124.8)	(30.9–48.2)	(10.6–25.9)
Fever at start of treatment, yes	11	7	3	1
Department of admission				
*Cardiology*	1	1	0	0
*Hematology*	7	7	0	0
*Infectious diseases*	4	1	2	1
*Internal medicine*	5	1	3	1
*Nephrology*	2	0	0	2
*Neurology*	1	1	0	0
*Oncology*	1	1	0	0
*Orthopedic*	12	10	1	1
*Respiratory medicine*	4	1	3	0
*Surgery*	2	1	1	0
*Urology*	1	1	0	0
Concomitant other antibiotic use	27	19	6	2
Length of hospital stay ^b^	10.0	11.0	12.0	10
	(7–20.3)	(7.5–28.0)	(7.0–21.8)	(5.5–10.5)

* Variables are listed as median (interquartile range (IQR)). ^a^ eGFR = estimated glomerular filtration rate calculated using the CKD-EPI (Chronic Kidney Disease Epidemiology Collaboration) formula. ^b^ Total length of hospital stay from day of admission to day of discharge.

**Table 3:** Parameter estimates of the structural, final model and bootstrap analysis.

Parameter	Structural Model		Final Model *		Bootstrap	
	Estimate	RSE (%) [Shrinkage (%)]	Estimate	RSE (%) [Shrinkage (%)]	Estimate	95% CI
CL (L/h)	4.50	11.7	3.74	9.80	3.74	3.03–4.41
V (L)	22.7	7.20	21.8	7.90	22.1	19.1–25.1
Interindividual variability						
CL (%CV)	78.9	20.3 [1.3]	31.3	29.6 [7]	31.1	22.6–38.7
V (%CV)	40.5	64.7 [21]	40.2	61.8 [18]	40.9	10.0–59.2
Residual variability						
Proportional error (%)	19.2	15.0	18.6	15.9	18.7	13.9–23.6
Covariates						
eGFR (CKD-EPI) (mL/min/1.73 m^2^) on CL	-	-	0.75	13.9	0.74	0.56–0.93
Concomitant antibiotic use on CL	-	-	1.56	12.4	1.57	1.20–1.94

* The equation of CL in the final model is: CL (L/h) = 3.74 × (CKDEPI/76.86)^0.75^ × 1.56^flag^; flag = 0 in case of no concomitant antibiotic use and flag = 1 in case of concomitant antibiotic use. Abbreviations: CL = clearance in L/hour, V = volume of distribution in L, RSE = relative standard error, 95% CI = 95% confidence interval, eGFR = estimated glomerular filtration rate, CKD-EPI= Chronic Kidney Disease Epidemiology Collaboration.

**Table 4:** Probability of PK/PD target attainment of 50% T > MIC for the first 24 h of treatment based on the observed data in the different renal function groups using different MIC values of common Gram-negative bacteria susceptible to ceftazidime as listed in the EUCAST.

	PTA (50% T_0–24_ > MIC)						
MIC (mg/L)	0.125	0.25	0.5	1	2	4	8
Renal Function Group							
Adequate renal function (eGFR ≥ 50 mL/min/1.73 m^2^, (n = 25))	100%	100%	100%	100%	100%	100%	100%
Moderate renal impairment (eGFR ≥ 30–50 mL/min/1.73 m^2^, (n = 10))	100%	100%	90%	90%	90%	90%	90%
Severe renal impairment (eGFR < 30 mL/min/1.73 m^2^, (n = 5))	100%	100%	100%	100%	100%	100%	100%

**Table 5:** Probability of PK/PD target attainment of 100% T > MIC for the first 24 h after the start of treatment based on the observed data in the different renal function groups using different MIC values of common Gram-negative bacteria susceptible to ceftazidime as listed in the EUCAST.

	PTA (100% T_0–24_ > MIC)						
MIC (mg/L)	0.125	0.25	0.5	1	2	4	8
Renal Function Group							
Adequate renal function (eGFR ≥ 50 mL/min/1.73 m^2^, (n = 25))	100%	100%	100%	100%	56%	48%	24%
Moderate renal impairment (eGFR ≥ 30–50 mL/min/1.73 m^2^, (n = 10))	90%	90%	90%	90%	90%	80%	50%
Severe renal impairment (eGFR < 30 mL/min/1.73 m^2^, (n = 4))	100%	100%	100%	100%	100%	100%	75%

**Table 6:** Probability of PK/PD target attainment of 50% T > MIC for 24–48 h after the start of treatment based on the observed data in the different renal function groups using different MIC values of common Gram-negative bacteria susceptible to ceftazidime as listed in the EUCAST.

	PTA (50% T_24–48_ > MIC)						
MIC (mg/L)	0.125	0.25	0.5	1	2	4	8
Renal Function Group							
Adequate renal function (eGFR ≥ 50 mL/min/1.73 m^2^, (n = 24))	100%	100%	100%	100%	100%	100%	100%
Moderate renal impairment (eGFR ≥ 30–50 mL/min/1.73 m^2^, (n = 10))	90%	90%	90%	90%	90%	90%	90%
Severe renal impairment (eGFR < 30 mL/min/1.73 m^2^, (n = 4))	100%	100%	100%	100%	100%	100%	100%

## Data Availability

Data available on request due to privacy restrictions.

## Supplementary Materials

The following supporting information can be downloaded at: https://www.mdpi.com/article/10.3390/antibiotics12030469/s1. Figure S1: Goodness of fit plots of the final model; File S1: Results: NONMEM control stream of the final model.
