# Supplementary material for: Pharmacokinetic/Pharmacodynamic Target Attainment of Ceftazidime in Adult Patients on General Wards with Different Degrees of Renal Function: A Prospective Observational Bicenter Cohort Study
Source: Antibiotics (Basel). 2023 Feb 25;12(3):469. doi: 10.3390/antibiotics12030469 (PMC10044023; doi:10.3390/antibiotics12030469)
Supplement: Supplementary file 1 [file antibiotics-12-00469-s001.zip › antibiotics-2203417-supplementary.pdf]

Supplementary Material

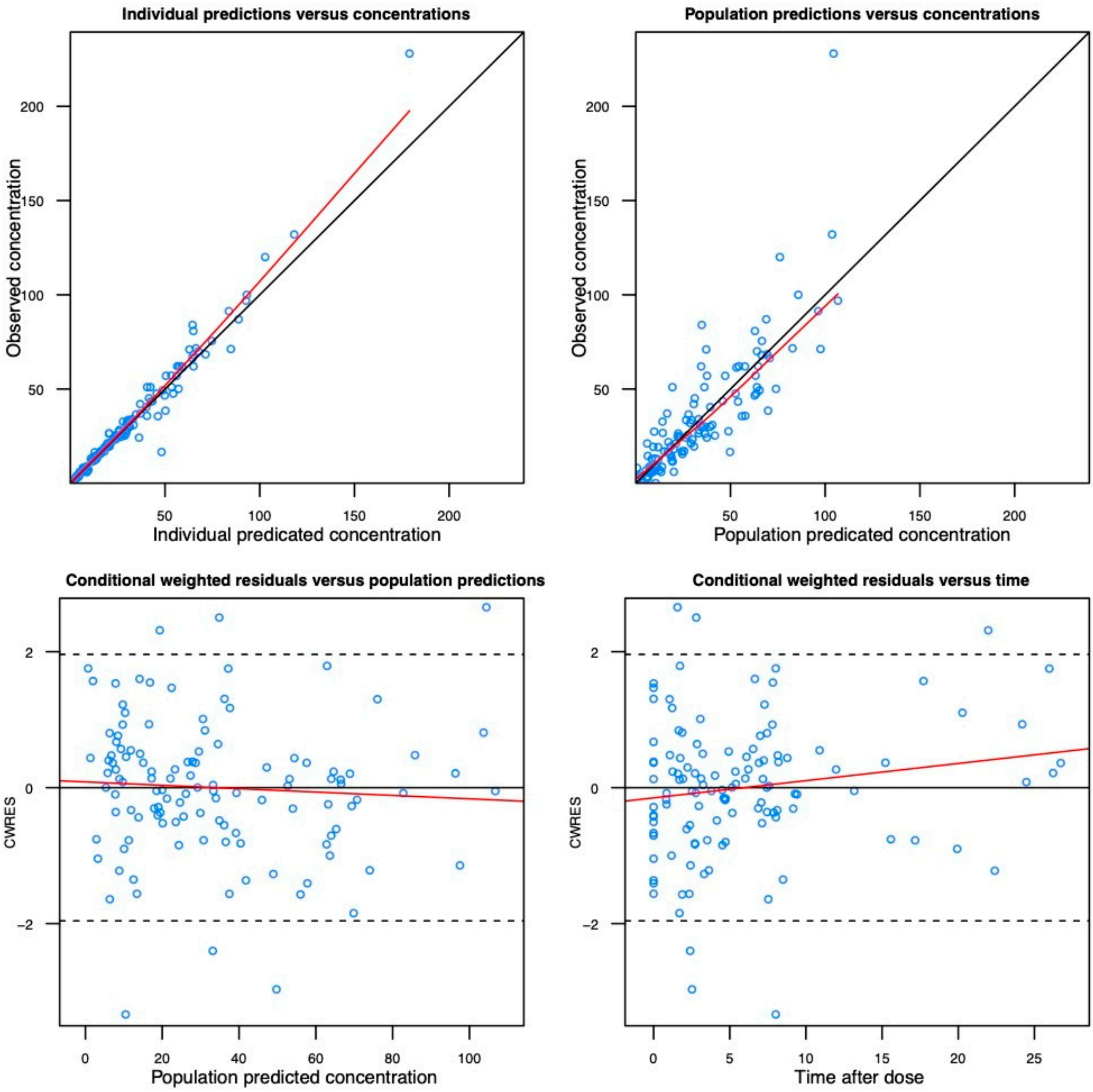

Figure S1 Goodness of fit plots of the final model. Individual predicted concentration versus (vs) observed concentration (left top), population predicted concentration vs observed concentration (right top), the conditional weighted residuals (CWRES) versus the population predicted concentration (left bottom) and CWRES versus the time after dose (right bottom). The data in the upper panels are evenly distributed around the line of identity (black solid line) and the data in the CWRES plots are evenly distributed around the x-axis both indicating no major bias in the final model. The red line is a smooth.

File S1:

Results: NONMEM Control stream of the final model

```
PROBLEM PK model
$INPUT ID DROP DROP DROP TIME TAD INDTAT RATE AMT DV DROP MDV
EVID WEIGHT IBW LBW HEIGHT BMI CREAT GENDER ETHNICITY AGE CRGT
MDRD CKDEPI EGFRCAT OCC FEVER DEPTORT DEPTHEM COMED
;---------------------------------
$DATA Dataset_TTCefta.csv IGNORE=#
$SUBROUTINES ADVAN1 TRANS2
$PK
FLAG1=0
IF(COMED.EQ.1)FLAG1=1
TVCL=THETA(3)*(CKDEPI/76.85)**THETA(4)* THETA(6)**FLAG1
CL=TVCL*EXP(ETA(1))
V = THETA(5) * EXP(ETA(2))
S1 = V
$THETA
(0.186)   ;1 proportional error
(0 fix)  ;2 ADDITIVE ERROR
(3.74)   ;3 CL
(0.75) ;4 est exponent TVCL effect CKDEPI
 (21.8) ;5 V
(1.56) ;6 effect concomittant AB$OMEGA
0.0936          ; IIV/BSV CL, fix to 0 to exclude
0.157           ; ETA V
$SIGMA
1 FIX    ;residual variability
$ERROR
IPRED = F
IRES = DV-IPRED
W = IPRED*THETA(1)+THETA(2)
IF (W.EQ.0) W = 1
IWRES = IRES/W
Y= IPRED+W*ERR(1)
$EST METHOD=1 INTERACTION
MAXEVAL=9999 SIG=3 PRINT=5 NOABORT POSTHOC
$COV PRINT=E UNCONDITIONAL
$TABLE ID TIME DV IPRED IWRES TAD AMT CWRES CL V ETA1 ETA2 CREAT
WEIGHT GENDER CKDEPI NOPRINT ONEHEADER FILE=sdtab038
```
